# Freshwater Macroalgae, *Oedogonium*, Grown in Wastewater Reduce Diet-Induced Metabolic Syndrome in Rats

**DOI:** 10.3390/ijms232213811

**Published:** 2022-11-09

**Authors:** Sunil K. Panchal, Naga K. R. Ghattamaneni, Marie Magnusson, Andrew Cole, David Roberts, Nicolas Neveux, Lindsay Brown, Nicholas A. Paul

**Affiliations:** 1 Functional Foods Research Group, University of Southern Queensland, Toowoomba, QLD 4350, Australia; 2 School of Science, Western Sydney University, Richmond, NSW 2753, Australia; 3 Te Aka Mātuatua—School of Science, University of Waikato, Tauranga 3112, New Zealand; 4 College of Marine & Environmental Sciences, James Cook University, Townsville, QLD 4811, Australia; 5 Pacific Biotechnologies Australia Pty Ltd., James Cook University, Townsville, QLD 4811, Australia; 6 School of Pharmacy and Medical Science, Griffith University, Gold Coast, QLD 4222, Australia; 7 School of Science, Technology and Engineering, University of the Sunshine Coast, Maroochydore, QLD 4558, Australia

**Keywords:** macroalgae, freshwater, biomass, rat model, high-carbohydrate, high-fat diet, metabolic syndrome

## Abstract

Macroalgae produce compounds with industrial, pharmaceutical and nutritional applications. In this study, biomass from the freshwater macroalgal genus *Oedogonium* was grown in either treated municipal wastewater (M) or ash dam water from a coal-fired power station (D). The biomass was investigated for its metabolic responses in high-carbohydrate, high-fat diet-fed rats, a model of human metabolic syndrome. The *Oedogonium* biomass cultured in M contained higher amounts of K, Mg, omega-3 polyunsaturated fatty acids (PUFA), insoluble fibre and β-carotene, while biomass grown in D contained higher amounts of Al, Fe, V, Zn, Mn and As. Biomass from M further increased body weight and inflammation in the heart and colon in high-carbohydrate, high-fat diet-fed rats. In contrast, biomass from D prevented changes in metabolic, cardiovascular and liver parameters without changing tissue histology. We suggest that increased intake of metals and metalloids through macroalgal biomass from D may decrease abdominal fat deposition while polysaccharides, PUFA and carotenoids from M may improve blood glucose responses in an obesogenic diet. Thus, macroalgal biomass grown in different wastewater sources could be acceptable for feed or food applications. This biomass could even provide potential health benefits in diet-induced metabolic syndrome.

## 1. Introduction

The diverse uses of seaweeds (marine macroalgae) throughout the world which include food sources for humans and livestock, medicines and building materials, have led to the proposal that humankind has been “saved by seaweeds”, especially in times of crises [1]. Extensive myths and legends are part of the background of current evidence-based research on seaweeds [2]. The nutritional benefits of algal-derived food products, predominantly from seaweeds, have been associated with their high-quality proteins, lipids, polysaccharides, vitamins and antioxidants [3,4]. Thus, seaweeds may be functional foods (defined as foods that provide health benefits along with nutrition) when consumed chronically in adequate amounts as part of a regular diet. Functional foods may be useful to treat or prevent metabolic syndrome [5,6], a cluster of risk factors that includes obesity, hypertension, dyslipidaemia and impaired glucose tolerance which increases the risk for development of cardiovascular disease, type two diabetes and some cancers [7]. We have reported that the diverse taxonomic groups of green [8,9], brown [10] and red [11,12] seaweeds are potential tropical functional foods for metabolic syndrome [13], but freshwater macroalgae have been rarely studied for their health benefits, potentially because commercial production is limited.

World-wide production of seaweeds was estimated at over 32 million tonnes in 2018 with 97.1% being farmed seaweeds [14]. Macroalgal forests in coastal ecosystems have been estimated to have an area of around 7 million km^2^ and a net primary production of 1.32 × 10^15^ g C/year [15]. Almost all seaweed production is from the sea, yet freshwater macroalgae lends itself to land-based production for industrial applications such as biofuel feedstock [16]. Strains of the freshwater genus *Oedogonium* (Chlorophyceae) are an ideal target for large-scale biomass production [17]. Further, cultivation of freshwater macroalgae is useful for the bioremediation of different sources of wastewater, where the growing biomass assimilates nutrients such as N, P, metals and trace metals, which are removed from the system when the biomass is harvested [18,19,20]. This then allows the reuse or discharge of clean freshwater, an increasingly rare resource [21], as well as a generation of new products from macroalgae such as biostimulants and soil conditioners [22,23,24]. Species of *Oedogonium* are effective in removing metals from the wastewater of commercial coal-fired power stations [25] and nutrients in municipal wastewater treatment plants [26,27,28]. Growing freshwater macroalgae for bioremediation of wastewater will likely produce biomass that is a potential functional food for metabolic syndrome as similar benefits have already been shown for marine macroalgal biomass.

This study characterises the chemical composition of *Oedogonium* biomass grown in two different wastewaters and reports the responses to each biomass in rats with diet-induced metabolic syndrome. Our first hypothesis was that *Oedogonium* biomass grown in contaminated wastewater from the ash dam of a coal-fired powerstation contained potentially harmful components which could induce toxic effects when fed to rats. In contrast, the biomass from treated municipal wastewater-grown macroalgae would contain valuable nutrients such as N, P, polysaccharides, fatty acids, carotenoids and trace metals. Our second hypothesis was that *Oedogonium* biomass cultivated in nutrient-rich municipal wastewater would improve the symptoms of diet-induced metabolic syndrome in rats, an accepted model of metabolic syndrome in humans [29].

## 2. Results

### 2.1. Composition of Macroalgae from Different Sources

Ash dam macroalgae (D) had up to five times greater amounts of Al, Ba and Mn, and more than five times greater amounts of Cd, Co, Cr, Cu, Fe, Mo, Ni, V and Zn compared to treated municipal wastewater-grown macroalgae (M). Treated municipal wastewater-grown macroalgae had up to three times greater content of Ca, K, Mg, Na, Pb and Sr (Table 1). The metalloids As and B were 80 times and 1.3 times greater in ash dam macroalgae, and likewise the non-metal S was 1.3 times higher in the *Oedogonium* cultivated in ash dam water. In contrast, P was 1.3 times greater in the *Oedogonium* cultivated in treated municipal wastewater. Ash dam macroalgae biomass had two-fold and 4.5-fold greater proportions of saturated and monounsaturated fatty acids, respectively, but 6.8-fold lower proportions of polyunsaturated fatty acids (Table 1). Ash dam macroalgae had 1.6 times and 1.7 times lower total dietary fibre and insoluble fibre, respectively, with minimal soluble fibre detected in either biomass. The concentrations of vitamins also differed between the two strains of *Oedogonium*; vitamin K_1_ was three times greater in the *Oedogonium* cultivated in the ash dam water, whereas β-carotene and vitamin B_12_ were 20 times and three times greater, respectively, in the *Oedogonium* cultivated in treated municipal wastewater (Table 1).

### 2.2. Metabolic Parameters and Body Composition

**Figure 1:**
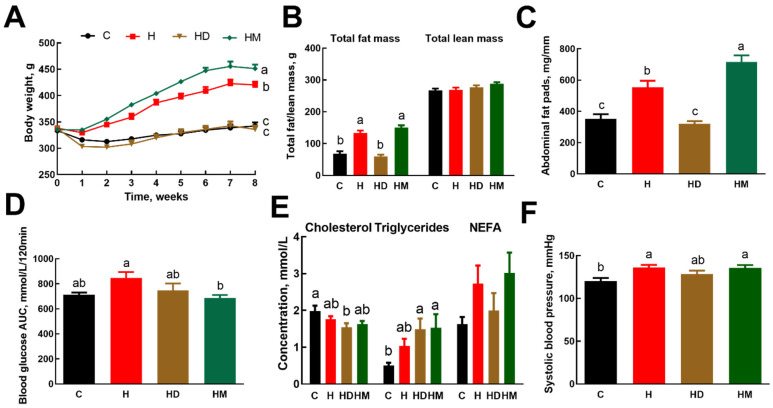
Effects of *Oedogonium* grown in ash dam water and treated municipal wastewater on (**A**) body weight, (**B**) whole-body fat and lean mass, (**C**) total abdominal fat pads, (**D**) blood glucose AUC during glucose tolerance test, (**E**) plasma lipid profile and (**F**) systolic blood pressure. Values are presented as means ± SEM (*n* = 8 rats per group). Means without a common letter are significantly different (a, b or c; *p* < 0.05). C, corn starch diet-fed rats; H, high-carbohydrate, high-fat diet-fed rats; HD, high-carbohydrate, high-fat diet-fed rats supplemented with *Oedogonium* grown in ash dam water; HM, high-carbohydrate, high-fat diet-fed rats supplemented with *Oedogonium* grown in treated municipal wastewater; AUC, area under the curve; NEFA, non-esterified fatty acids.

High-carbohydrate, high-fat (H) diet-fed rats had increased body weight, whole-body fat mass, abdominal fat pads and systolic blood pressure compared to corn starch (C) diet-fed rats (Figure 1). Treated municipal wastewater-grown macroalgae increased body weight in H rats fed treated municipal wastewater-grown *Oedogonium* biomass (HM) (Figure 1A; Table 2) with no change in fat or lean mass compared to H rats (Figure 1B). In contrast, ash dam macroalgae prevented increases in body weight in H rats fed ash dam *Oedogonium* biomass (HD) (Figure 1A; Table 2) and body fat (Figure 1B) so that these parameters were similar to C rats and markedly lower than H rats (Figure 1B); however, the lean mass remained unchanged compared to both C and H rats (Figure 1B). These changes were observed without any changes in food, water or energy intakes compared to H rats, hence decreasing feed efficiency in HD rats to zero while increasing it in HM rats compared to H rats (Table 2). Treated municipal wastewater-grown macroalgae increased total abdominal and retroperitoneal fats with no change in epididymal and omental fats (Figure 1C; Table 2). In contrast, ash dam macroalgae prevented the increase in total abdominal, retroperitoneal, epididymal and omental fats in HD rats. Bone mineral density and bone mineral content were decreased in HD rats but were unaffected in HM rats compared to H rats (Table 2).

Biomass from both water sources reduced basal blood glucose concentrations and blood glucose concentrations at 120 min compared to H rats. The area under the curve for HD rats was intermediate to H and HM rats with HM rats having lower area under the curve compared to H rats (Figure 1D; Table 2). Plasma lipid concentrations were unaffected by dietary macroalgae (Figure 1E).

### 2.3. Liver, Heart and Gastrointestinal Parameters

**Figure 2:**
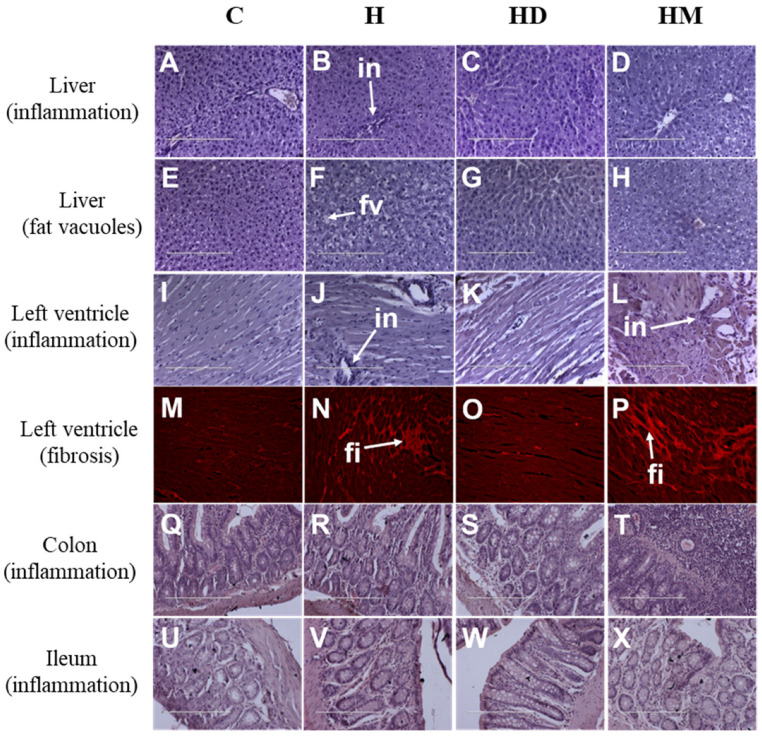
Effects of *Oedogonium* grown in ash dam water and treated municipal wastewater on inflammation and fibrosis in the heart, colon and ileum structure of rats (*n* = 4 rats per group). Hematoxylin and eosin staining of the liver showing inflammatory cells (*in*, arrow head) and fat vacuoles (*fv*, arrow head) in C (**A**,**E**); H (**B**,**F**); HD (**C**,**G**) and HM (**D**,**H**) rats. Hematoxylin and eosin staining and picrosirius red staining of the left ventricle showing inflammatory cells (*in*, arrow head) and fibrosis (*fi*, arrow head), respectively, in C (**I**,**M**), H (**J**,**N**), HD (**K**,**O**) and HM (**L**,**P**) rats. Hematoxylin and eosin staining of the colon and ileum showing inflammatory cells in C (**Q**,**U**), H (**R**,**V**), HD (**S**,**W**) and HM (**T**,**X**) rats. Scale bar, 100 μm. C, corn starch diet-fed rats; H, high-carbohydrate, high-fat diet-fed rats; HD, high-carbohydrate, high-fat diet-fed rats supplemented with *Oedogonium* grown in ash dam water; and HM, high-carbohydrate, high-fat diet-fed rats supplemented with *Oedogonium* grown in treated municipal wastewater.

Liver wet weights of both treated groups were similar to H rats and higher than C rats (Table 2). Plasma activities of alanine transaminase and aspartate transaminase were unchanged in both HM and HD rats (Table 2). Liver B content was very low in H rats compared to C rats and both macroalgae-treated groups had intermediate B content compared to C and H rats (Table 3). Liver V content was similar in C, H and HM rats; ash dam macroalgae markedly increased V content in the liver of HD rats compared to other groups (Table 3). Livers from HD and HM rats showed an absence of inflammation (Figure 2C,D) and steatosis (Figure 2G,H), unlike livers from H rats which showed inflammatory cell infiltration and steatosis (Figure 2B,F).

Systolic blood pressure was unchanged in HM and HD rats compared to H rats (Figure 1F). Left and right ventricular weights were unchanged by supplementation with macroalgae whereas kidney and spleen weights were increased in HM rats compared to H rats (Table 2). HM (Figure 2L,P) rats had higher inflammation and fibrosis in the left ventricle compared to H rats (Figure 2J,N) whereas HD rats (Figure 2K,O) had lower inflammation and fibrosis in the left ventricle compared to H rats (Figure 2J,N). The colon and ileum from HM rats showed higher inflammation than other groups whereas the ileum from HD rats showed no abnormalities (Figure 2S,W) unlike the colon (Figure 2T,X). Table 2 and Table 3 provide other metabolic and tissue variables including metal contents in the liver.

## 3. Discussion

Malnutrition, including obesity, undernutrition and other dietary risks, is now the leading cause of poor health in the world [30]. Overnutrition and obesity are major challenges in both developed and developing countries [31]. The consumption of energy-rich foods has increased with most of the energy coming from simple sugars and saturated fats, leading to a higher prevalence of obesity [32]. The risk of developing cardiovascular disease, type 2 diabetes and cancers also increases with obesity [33]. The greatest burden of malnutrition in low- and middle-income countries comes from the combination of the high prevalence of stunting (28%), wasting (8.8%) and underweight (17.4%) with increasing overweight in children less than five years of age [30]. The Lancet Commission in 2019 reported economic losses of about $3.5 trillion annually from malnutrition, equivalent to 11% of the GDP in Africa and Asia [30].

Seaweeds have been an essential part of human diets [1], possibly providing the nutritional and energetic requirements for the unique development of a large human brain [34] and are now consumed mainly in East Asia [35,36]. Freshwater macroalgae have received little attention as functional foods for metabolic syndrome compared to seaweeds or microalgae [4]. It is feasible that functional foods from freshwater macroalgae could provide solutions to both over- and under-nutrition. As an example, the freshwater macroalgae *Spirogyra varians* can provide a viable source of nutrition in human diets [37].

This study showed that the biomass of a freshwater macroalgae, *Oedogonium*, cultivated in ash dam wastewater containing increased concentrations of metals and metalloids prevented obesity development in rats fed a high-carbohydrate, high-fat diet. This response was manifested as a prevention of increases in body weight, whole-body fat mass and abdominal fat together with improvements in blood glucose tolerance. Ash dam macroalgae prevented inflammation in the heart and colon, steatosis in the liver and fibrosis in the heart. These changes occurred without obvious toxicity, despite the accumulation of metals and metalloids in the macroalgal biomass and in the rat liver after the consumption of the macroalgae. While the consumption of *Oedogonium* cultivated in treated municipal wastewater also resulted in improvements in blood glucose tolerance and decreased steatosis in the liver, these changes were accompanied by increased body weight and abdominal fat, inflammation in the heart and colon, and fibrosis in the heart. These results clearly distinguish the metabolic responses of the rat model to two different sources of macroalgae with notably differentiated biochemical profiles.

Metabolic syndrome in humans is characterised by extensive changes in the gut microbiota with decreased capacity to metabolise carbohydrates and short-chain fatty acids. This decrease may be reversed with increased dietary prebiotic fibre [38]. The complex polysaccharides in seaweeds may be a viable nutritional source of the prebiotics to reverse changes in the gut microbiota [39]. The prebiotic role of these macroalgal polysaccharides, mostly from seaweed, and their health benefits have been widely discussed but clinical trials are still necessary [40]. While almost all investigations have been carried out on marine macroalgae, the freshwater green macroalgae, *Cladophora surera*, synthesised sulphated polysaccharides similar to the marine species of this genus [41]. However, the increased insoluble fibre in the treated municipal wastewater biomass (37.4%) compared to ash dam wastewater biomass (21.4%) does not correlate with the greater effectiveness of the ash dam biomass in the prevention of increased abdominal fat deposition.

Trace elements are involved in the development and treatment of obesity. They are chemical micronutrients including Cr, Co, Cu, Fe, Mn, Se and Zn that are needed in very small amounts, yet have a prominent role in the homeostasis of physiological and metabolic processes in our body [42]. Seaweeds may accumulate heavy metals and thus induce toxicity in humans when consumed, albeit rarely [43]. This is consistent with studies with freshwater macroalgae showing that *Oedogonium* (Genbank Accession Number KF606974) cultured with flue gas reduced the concentrations of all metals in wastewater from a coal-fired power station [25], that *Oedogonium westii* effectively removed Cd, Ni, Cr and Pb ions from aqueous systems [44], and that the freshwater macroalgae, *Cladophora fructa*, removed Cu, Zn, Cd and Hg ions from aqueous solutions [45]. However, trace metals found in industrial wastewater including Cr and V may be beneficial to human health and disease [46,47,48,49]. Their importance in reducing diet-induced obesity has been shown by improved insulin signalling and glucose metabolism [46,49,50]. In high-fat diet-fed female Wistar rats, decreased adipose tissue content of Cr and V correlated with an increase in adipose tissue endocrine dysfunction [49]. Decreased content of these trace elements in adipose tissue due to caloric excess has been hypothesised to lead to the development of adipose tissue insulin resistance through disruption of intra-adipocyte insulin signalling, and further adipokine imbalance that leads to obesity [50]. Along with decreased insulin resistance, Cr and Zn also decrease inflammation, which is the link between insulin resistance and obesity that leads to metabolic syndrome [50].

The trace elements in macroalgae when consumed as part of the diet could play a role in the control of metabolic syndrome risk factors in places where these dietary nutrients are missing or where traditional tillage cropping has removed many of the minerals from the soil. Zn produces insulinomimetic effects and improved glucose tolerance and ash dam macroalgae had high amounts of Zn [51]. Na and K were present in high amounts in comparison to other minerals and, though there was no change in systolic blood pressure, these minerals play roles in managing hypertension [52]. Trace elements such as Cr, Se and V when taken in optimal doses can improve the symptoms of metabolic syndrome with their insulinomimetic effects [53]. Cr administration lowered adipose tissue content in obese rats compared to control rats [49], and supplementation of Cr histidinate lowered body weight and serum glucose with increased Nrf2 expression and decreased NF-κB expression [54]. Se deficiency can cause heart failure [55] and its supplementation showed cardioprotective effects [56]. VO (dmpp)2, an oxovanadium, administered to fat rats reduced obesity and hepatic triglycerides [57]. High-fat diet-fed C57BL/6 mice treated with V-rich groundwater had reduced obesity which may be due to the inhibition of preadipocyte differentiation by decreasing the peroxisome proliferator-activated receptor γ (PPAR-γ) and CCAAT-enhancer-binding protein expression [58]. Ash dam biomass had higher contents of these three trace elements than the treated municipal wastewater biomass and supports their role in the prevention of obesity in rats treated with ash dam grown *Oedogonium* biomass. Inorganic As has been established as a carcinogen for humans with increased risk of certain cancers and other metabolic disease [59,60,61]. Many foods can accumulate high levels of inorganic As and can introduce these into the food cycle [59]. Organic As, on the other hand, has been considered to be non-toxic [62,63]. In this study, higher amounts of trace metals such as As, V and Se were absorbed into the macroalgae (10 to 100 times), but the relative difference between the concentrations in the rat liver was much lower than that between the algae. This may suggest that the excess amount of these metals including As may not be absorbed and accumulated from macroalgae in the rats.

Omega-3 fatty acids and carotenoids are present in macroalgae, although at lower concentrations. These components have proven health benefits associated with them [64,65,66,67], but their lower concentrations in macroalgae may not provide sufficient doses for physiological responses. However, these compounds may provide benefits to complement responses from other components.

One of the limitations of our study is the lack of a macroalgae intervention to corn starch diet-fed rats, as our primary aim was to investigate effects of macroalgae in obesity prevention. Although both interventions showed no signs of toxicity in high-carbohydrate, high-fat diet-fed rats, the high content of metals and metalloids in biomass cultured in ash dam water could exhibit some toxic effects in corn starch diet-fed rats or other basal diets more broadly. Further studies on feeding macroalgae interventions to corn starch diet-fed rats would identify any such effects. Moreover, a reversal protocol of feeding macroalgae to both corn starch diet-fed rats and high-carbohydrate, high-fat diet-fed rats would provide responses that can be compared with our reversal protocol studies with seaweeds showing beneficial responses [8,9,10,11,12].

Thus, our first hypothesis that *Oedogonium* biomass grown in ash dam water would be toxic has now been shown to be incorrect as this biomass prevented most of the signs of diet-induced metabolic syndrome in rats without signs of gastrointestinal, liver or heart toxicity. Further, our second hypothesis that *Oedogonium* biomass grown in municipal wastewater would improve the symptoms of diet-induced metabolic syndrome in rats was shown to be valid only for the prevention of diet-induced hyperglycaemia. Based on the results in our study, it would be interesting to analyse how these macroalgae impact gut microbiota.

## 4. Materials and Methods

### 4.1. Sources of Freshwater Macroalgae

Two strains of the freshwater macroalgal genus *Oedogonium* were cultured for this study. The first strain (*Oedogonium intermedium*, Genbank Accession number: KF606977 [68]) was originally collected from an agricultural irrigation channel at Brandon, QLD, Australia (19°39′ S, 147°24′ E) [69]. This strain was maintained in stock cultures at the Marine and Aquaculture Facilities, James Cook University, Townsville, QLD, Australia prior to culture in the primary treated municipal wastewater from Cleveland Bay Wastewater purification plant in Townsville. Cultures were maintained at James Cook University in 3 × 10,000 L tanks with a water exchange (new for old) of 800 L every day over a 4-week period [27]. The biomass yield over the culture period ranged from 6.8 to 9.9 g DW m^−2 ^d^−1^ (equivalent to ~30 tonnes per hectare per year). A second strain (*Oedogonium* sp., Genbank Accession number: KF606974 [70]) was isolated from the 46,000 ML ash dam of Tarong Power station, a 700 MW coal-fired power station located at Tarong, QLD, Australia (26°46′ S, 151°54′ E). This strain was cultured in 3 × 10,000 L tanks on site at the power station using ash dam water amended with flue gas from the combustion of coal, with 5000 L exchanged every three days throughout the 4-week culture period [25]. The biomass yield over the culture period ranged from 2.9 to 8.2 g DW m^−2 ^d^−1^ (equivalent to ~20 tonnes per hectare per year). Biomass from each water source was collected weekly over the 4-week culture period and a pooled blended sample was used for assays and biochemical analysis. Biomass was air-dried and stored at room temperature until use. Ash dam macroalgae was labelled as D and municipal waste treatment plant macroalgae was labelled as M. The two *Oedogonium* strains were closely related (Clade Tar1 [KF606974] and Tsv2 [KF606977] with a bootstrap value of 88%; we cannot state that these are of the same species [69]). Dried biomass was used for all biochemical analyses and for diet inclusion in the live rat study.

### 4.2. Chemical Analysis of Macroalgae

The full biochemical profiles of biomass from each wastewater source were determined by analysis for proximate components (lipid, protein, carbohydrate, ash, moisture and dietary fibre) and element concentrations (C, H, O, N, S and metal/metalloid/halogens), as well as for specific nutritional compounds (antioxidants, vitamins and pigments).

Total lipids were quantified in a 200.0 ± 0.1 mg subsample using solvent extraction [71]. Fatty acids were extracted and transesterified from a separate subsample of biomass (30.0 ± 0.1 mg) following a one-step extraction/transesterification method (methanol/acetyl chloride; 95:5 *v*/*v*) [72]. Fatty acid methyl esters were separated and quantified by gas chromatography–mass spectrophotometry on an Agilent 7890c GC/5975c EIMS system equipped with a DB-23 capillary column (cyanopropyl stationary phase [60 m × 0.25 mm id × 0.15 μm], Agilent Technologies, Mulgrave, VIC, Australia) [72]. The quantity of fatty acids was determined by the comparison of peak areas of external standards (Sigma Aldrich, Castle Hill, NSW, Australia) and was corrected for recovery of internal standard (heptadecanoic acid, C17:0).

Protein was determined as the sum of amino acids based on a quantitative amino acid analysis (16 amino acids) performed at Australian Proteome Analysis Facility (APAF) at Macquarie University, Sydney, NSW, Australia, while carbohydrate was calculated by difference as 100 – ∑ (lipid, protein, ash, moisture) where lipids, proteins, ash and moisture are expressed as a percentage of the total mass. Moisture content was measured on a minimum of 1.0 g biomass at 105 °C to constant weight (MS-70 moisture analyser, A&D Company Ltd., Toshima, Tokyo, Japan). The same biomass sample was then weighed to 1 mg precision followed by combustion in air (550 °C, 6 h; SEM muffle furnace, LabTek, Brendale, QLD, Australia) to determine ash content per gram dry weight.

The total dietary fibre content, including soluble and insoluble components that comprise the main forms of carbohydrates, was analysed on a 10 g sample, following standard methods (AOAC Official Method 985.29 total dietary fibre in foods, and AOAC Official Method 993.19 soluble dietary fibre in food and food products) by Grain Growers Ltd. (Sydney, Australia; now Australian Export Grains Innovation Centre (AEGIC)).

A subsample (200 mg) of each biomass source was also analysed for the contents of C, N, H and S (OEA Laboratory Ltd., United Kingdom, http://www.oealabs.com) using GC-TCD. The content of oxygen was calculated by difference as %O = 100 – ∑(C, H, N, S, ash) where C, H, N, S and ash are expressed as a percentage of the total mass. Gross energy content (kJ/g) of the biomass expressed as the higher heating value was then calculated [73] based on CHONS elemental composition and ash content. The content of 24 elements in the biomass was measured by Inductively Coupled Plasma Mass Spectrometry (ICP/MS) (Varian 820-MS, Varian, Belrose, NSW, Australia) at the Advanced Analytical Centre, James Cook University [74].

A further series of subsamples was taken for the following nutritional analyses. Vitamins were quantified by HPLC (ascorbic acid and vitamin K_1_) and by the *Euglena gracilis* microbiological assay (vitamin B_12_) at the National Measurement Institute, Sydney, NSW, Australia. The pigments chlorophyll a and b were extracted in HPLC-grade methanol and quantified spectrophotometrically (SpectroStar nano, BMG LabTech, Mornington, VIC, Australia) [75], while α- and β-carotene were quantified by HPLC at the National Measurement Institute following standard protocols [76].

### 4.3. Studies in Live Rats

**Figure 3:**
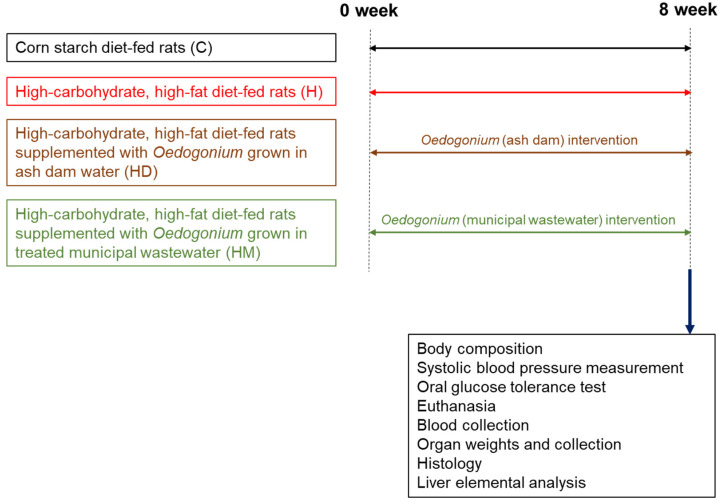
Study design to characterise responses to *Oedogonium* biomass in rats with diet-induced metabolic syndrome.

Male Wistar rats (8–9 weeks old, 330–340 g, *n* = 32) were obtained from the Animal Resources Centre, Murdoch, WA, Australia. Rats were randomly divided into four groups (Figure 3). Control groups were fed either a corn starch diet (C; *n* = 8) or a high-carbohydrate, high-fat diet (H; *n* = 8) [29]. Two groups were fed a high-carbohydrate, high-fat diet containing 5% dried biomass of freshwater macroalgae grown in ash dam water or treated municipal wastewater (HD and HM) to replace 5% water in these diets (*n* = 8 in each group). These diets were given to the rats for eight weeks. Drinking water for high-carbohydrate, high-fat diet-fed rats was supplemented with 25% fructose, whereas corn starch diet-fed rats were given drinking water without any additive. The composition of C and H diets is described in our previous studies [29,77]. All the rats were individually housed in temperature-controlled 12-h light/dark conditions and were given *ad libitum* access to food and water.

Body weight and intakes of food and water were measured daily for all rats. Abdominal circumferences were measured using a standard measuring tape under light sedation with Zoletil (tiletamine 10 mg/kg, zolazepam 10 mg/kg, i.p.; Virbac, Peakhurst, NSW, Australia) [29]. Energy intakes (including the values for D—14.77 kJ/g; M—17.66 kJ/g) and feed efficiency were calculated from daily food intakes [29]. Body composition was measured using dual-energy X-ray absorptiometry at the end of the protocol using a Norland XR36 DXA instrument (Norland Corp., Fort Atkinson, WI, USA) under light anaesthesia with Zoletil (tiletamine 10 mg/kg and zolazepam 10 mg/kg, i.p.) and Ilium Xylazil (xylazine 6 mg/kg, i.p.). Scans were analysed using the manufacturer’s recommended software for use in laboratory animals (Small Subject Analysis Software, version 2.5.3/1.3.1; Norland Corp.) [29].

Systolic blood pressures of rats were measured at 0 and 8 week periods of the protocol under sedation with Zoletil (tiletamine 10 mg/kg, zolazepam 10 mg/kg, i.p.), using an MLT1010 Piezo-Electric Pulse Transducer (ADInstruments, Sydney, NSW, Australia) and an inflatable tail cuff connected to a MLT844 Piezo-Electric Pressure Transducer (ADInstruments) and PowerLab data acquisition unit (ADInstruments) [29].

At eight weeks of the protocol, rats were deprived of food overnight for 12 h for oral glucose tolerance testing. During this food deprivation period, fructose-supplemented drinking water was replaced with normal drinking water. Oral glucose tolerance tests were performed after determining basal blood glucose concentrations in tail vein blood using Medisense Precision Q.I.D. glucose meters (Abbott Laboratories, Bedford, MA, USA). Rats were given a glucose load of 2 g/kg body weight as 40% glucose solution by oral gavage and blood glucose concentrations were measured at 0, 30, 60, 90 and 120 min after oral glucose administration [29]. Blood glucose concentrations over the period of 120 min were used to calculate the area under the curve.

### 4.4. Studies after Euthanasia

Rats were euthanised with Lethabarb (pentobarbitone sodium, 100 mg/kg, i.p.; Virbac). After the induction of euthanasia, heparin (200 IU; Sigma-Aldrich, Bayswater, Australia) was injected through the right femoral vein. The abdomen was then opened, and blood (~5 mL) was withdrawn from the abdominal aorta and collected into heparinised tubes. Blood was centrifuged at 5000× *g* for 15 min to obtain plasma. Plasma concentrations of total cholesterol and triglycerides and plasma activities of alanine transaminase and aspartate transaminase were determined using kits and controls supplied by Olympus using an Olympus analyser (AU 400, Tokyo, Japan) [29]. Nonesterified fatty acids in plasma were determined using a commercial kit (Wako, Osaka, Japan) [29].

After blood withdrawal, hearts were perfused to clear the blood and separated into left ventricle (with septum) and right ventricle and weighed. The liver, kidneys, spleen, retroperitoneal fat, epididymal fat and omental fat were removed separately and weighed. These organ weights were normalised against the tibial length at the time of organ removal and expressed as mg/mm of tibial length [29].

The heart, liver, ileum and colon were removed from the rats (*n* = 5 from each group) within five minutes after euthanasia and these tissues were fixed in 10% neutral buffered formalin. The samples were then dehydrated and embedded in paraffin wax. Thin sections (5 µm) of these tissues were cut and stained with hematoxylin and eosin to study infiltration of inflammatory cells. Heart tissues were also stained with picrosirius red to study collagen deposition. Liver tissues were stained for fat vacuoles with hematoxylin and eosin stain [29].

Livers collected after euthanasia were stored at −20 °C until analysis. Liver samples were thawed and homogenised using an automated homogeniser (Miltenyi Biotec, San Diego, CA, USA). Samples were then dried at 60 °C and used for quantification of 24 elements by the Advanced Analytical Centre at James Cook University using ICP/MS.

### 4.5. Statistical Analysis

Macroalgae composition values are presented as mean ± standard deviation (SD), *n* = 3 (replicate subsamples of the biomass used for the rat component were taken to characterise the biochemical properties, not for formal comparison between the two water sources). Values from rat measurements are presented as mean ± standard error of the mean (SEM), *n* = 8. Differences between the groups were determined by one-way analysis of variance. Variables with statistically significant differences were treated with Newman–Keuls post hoc test to compare all groups of animals. Statistical analyses were performed using GraphPad Prism version 9.4 for Windows. A *p* value of <0.05 was considered as statistically significant.

## 5. Conclusions

The freshwater macroalgae, *Oedogonium*, cultivated in ash dam water had a markedly different biochemical profile to that cultivated in treated municipal wastewater with elevated levels of metals and trace elements, including Al, Fe, V, Zn, Mn and As. These metals and trace elements contributed to the prevention of obesity and the symptoms of metabolic syndrome as measured in rats fed a high-carbohydrate, high-fat diet with *Oedogonium* cultivated in ash dam water. Biomass cultivated in treated municipal wastewater failed to prevent obesity but helped in improving other metabolic parameters. This study supports the capacity to cultivate freshwater macroalgae with targeted biochemical profiles in selected or modified water sources, such that the biomass provides nutraceutical benefits in mitigating obesity and the symptoms of metabolic syndrome.

## Figures and Tables

**Table 1:** Elemental and macronutrient composition of macroalgae.

	*Oedogonium* Cultivated in Ash Dam Water (D)	*Oedogonium* Cultivated in Treated Municipal Wastewater (M)
Elements and proximate composition (% dry weight)		
C	32.41 ± 0.29	38.90 ± 0.05
H	5.31 ± 0.14	6.09 ± 0.01
O	19.64 ± 0.024	24.03 ± 1.68
N	5.15 ± 0.02	5.37 ± 0.11
S	0.33 ± 0.04	0.30 ± 0.015
F	0.035 ± 0.0002	0.0006 ± 0.0
Cl	0.209 ± 0.002	0.814 ± 0.012
Br	0.0032 ± 0.0001	0.0021 ± 0.00005
I	0.005 ± 0.0001	<0.0016
Moisture	5.16 ± 0.35	6.41 ± 1.38
Ash	32.00 ± 0.06	18.90 ± 0.20
Protein (sum of amino acids)	21.44	23.87
Total lipids	3.40 ± 0.25	4.82 ± 0.24
Carbohydrate (by difference)	37.99	46.01
Metals, non-metals and metalloids (mg/kg dry weight)		
Al	1315.0 ± 35.4	276.5 ± 2.1
As	99.95 ± 1.49	1.26 ± 0.014
B	9.61 ± 0.16	7.63 ± 0.09
Ba	209.5 ± 0.71	77.85 ± 0.50
Ca	9010 ± 141	14,450 ± 212
Cd	2.20 ± 0.02	0.11 ± 0.009
Co	5.75 ± 0.099	0.84 ± 0.008
Cr	16.35 ± 0.071	1.32 ± 0.04
Cu	48.70 ± 0.85	13.35 ± 0.21
Fe	14,500 ± 141	423.5 ± 6.4
Hg	<0.5	<0.5
K	10,550 ± 71	28,400 ± 1839
Mg	2485 ± 35	6165 ± 35
Mn	737.0 ± 9.9	184.0 ± 2.8
Mo	10.10 ± 0.00	0.82 ± 0.38
Na	1550 ± 14	1990 ± 14
Ni	42.60 ± 0.57	2.05 ± 0.02
P	10,900 ± 0	13,900 ± 141
Pb	0.83 ± 0.016	2.15 ± 0.134
S	3975 ± 219	3080 ± 141
Se	13.60 ± 0.28	<1
Sr	124.50 ± 0.71	180.5 ± 0.71
V	723.0 ± 4.24	0.82 ± 0.02
Zn	258.0 ± 4.24	42.15 ± 1.91
Fatty acids (% of total fatty acids)		
C14:0	9.08 ± 0.35	0.91 ± 0.01
C15:0	0.44 ± 0.02	0.12 ± 0.01
C16:0	50.84 ± 0.27	26.50 ± 0.09
C16:1*n*-7	22.90 ± 0.38	4.69 ± 0.13
C16:2*n*-6	0.11 ± 0.06	0.94 ± 0.04
C17:0	0.26 ± 0.09	0.32 ± 0.04
C16:3*n*-3	0.59 ± 0.38	18.28 ± 0.30
C18:0	1.23 ± 0.04	0.80 ± 0.01
C18:1*n*-9	5.24 ± 0.21	1.75 ± 0.04
C18:2*n*-6	1.90 ± 0.07	4.59 ± 0.04
C18:3*n*-6	ND	2.97 ± 0.10
C18:3*n*-3	5.63 ± 0.05	30.82 ± 0.18
C18:4*n*-3	ND	3.81 ± 0.05
C20:1*n*-9	0.62 ± 0.05	ND
C20:2*n*-6	1.20 ± 0.18	0.45 ± 0.10
C20:3*n*-6	ND	1.99 ± 0.07
C22:0	ND	1.05 ± 0.06
Total SFA	61.85 ± 0.54	29.71 ± 0.06
Total MUFA	28.75 ± 0.41	6.44 ± 0.12
Total PUFA	9.40 ± 0.49	63.85 ± 0.17
Total *n*-3 PUFA	6.22 ± 0.35	52.90 ± 0.40
Total *n*-6 PUFA	3.18 ± 0.26	10.95 ± 0.26
*n*-3:*n*-6	5.63 ± 0.05	30.82 ± 0.18
Dietary fibre (% dry weight)		
Total dietary fibre	23.8	35.3
Insoluble fibre	21.4	34.5
Vitamins		
α-carotene (μg/100 g)	<5	<5
Ascorbic acid (mg/100 g)	<1	<1
β-carotene (μg/100 g)	320	6400
Vitamin K_1_ (μg/100 g)	37	12
Vitamin B_12_ (μg/100 g)	218	634
Chlorophyll (% dry weight)		
Chlorophyll *a*	0.20 ± 0.01	0.59 ± 0.01
Chlorophyll *b*	0.50 ± 0.01	1.43 ± 0.04
Amino acids (mg/g dry weight)		
Histidine	3.7	4.6
Serine	10.5	12.3
Arginine	14.5	14.3
Glycine	12.6	13.0
Aspartic acid	24.8	28.7
Glutamic acid	28.2	32.1
Threonine	11.9	13.2
Alanine	15.1	16.5
Proline	10.3	12.0
Lysine	12.9	15.0
Tyrosine	8.3	9.6
Methionine	4.0	4.1
Valine	14.3	15.9
Isoleucine	10.9	11.3
Leucine	19.9	21.9
Phenylalanine	12.5	14.2

Values are mean ± SD, *n* = 3 for each group where replicate samples were analysed. Three subsamples of both macroalgae biomass used in the rat feeding trial were taken for biochemical analysis. Biomass was milled and mixed thoroughly prior to analysis and use in the feeding trial. Standard deviation for each analysis is provided to inform the consistency of the variable. No statistical comparisons were made between the biomass as the subsamples were not independent. SFA, saturated fatty acids; MUFA, monounsaturated fatty acids; PUFA, polyunsaturated fatty acids; ND, not detected.

**Table 2:** Effects of macroalgae on metabolic, tissue and biochemical variables.

Variables	C	H	HD	HM
Body weight gain, g	17.0 ± 7.0 ^c^	81.0 ± 5.0 ^b^	−1.0 ± 5.6 ^c^	124.0 ± 6.6 ^a^
Food intake, g/day	36.7 ± 1.3 ^a^	21.7 ± 0.4 ^b^	22.8 ± 1.5 ^b^	22.6 ± 0.4 ^b^
Water intake, g/day	28.9 ± 2.5	20.3 ± 1.6	24.2 ± 1.8	26.4 ± 1.3
Energy intake, kJ/day	411.6 ± 14.6 ^b^	465.7 ± 7.6 ^a^	515.6 ± 28.5 ^a^	524.8 ± 10.0 ^a^
Feed efficiency, g/kJ	0.04 ± 0.02 ^c^	0.17 ± 0.01 ^b^	0.00 ± 0.01 ^d^	0.24 ± 0.01 ^a^
Abdominal circumference, cm	18.2 ± 0.3 ^c^	21.1 ± 0.2 ^b^	18.6 ± 0.3 ^c^	22.4 ± 0.2 ^a^
Bone mineral density, g/cm^2^	0.163 ± 0.003 ^ab^	0.170 ± 0.004 ^a^	0.158 ± 0.003 ^b^	0.172 ± 0.002 ^a^
Bone mineral content, g	10.0 ± 0.3 ^b^	11.8 ± 0.3 ^a^	9.3 ± 0.3 ^b^	12.6 ± 0.2 ^a^
Basal blood glucose concentrations (week 8), mmol/L	4.1 ± 0.3 ^b^	5.2 ± 0.2 ^a^	4.5 ± 0.2 ^b^	4.3 ± 0.1 ^b^
Blood glucose 120 min (week 8), mmol/L	4.7 ± 0.2 ^b^	6.2 ± 0.4 ^a^	4.7 ± 0.4 ^b^	4.5 ± 0.2 ^b^
Retroperitoneal fat, mg/mm	154.0 ± 13.3 ^c^	253.8 ± 26.7 ^b^	130.1 ± 10.6 ^c^	338.9 ± 24.9 ^a^
Epididymal fat, mg/mm	89.7 ± 8.1 ^c^	131.2 ± 10.5 ^b^	70.4 ± 3.6 ^c^	182.5 ± 15.3 ^a^
Omental fat, mg/mm	108.3 ± 11.2 ^b^	169.6 ± 8.4 ^a^	120.6 ± 9.1 ^b^	194.4 ± 9.9 ^a^
Left ventricle + septum, mg/mm	18.6 ± 0.7 ^b^	20.0 ± 0.8 ^ab^	19.2 ± 0.9 ^b^	22.1 ± 0.7 ^a^
Right ventricle, mg/mm	4.25 ± 0.19	4.08 ± 0.46	3.40 ± 0.22	4.38 ± 0.32
Liver, mg/mm	223 ± 10 ^b^	279 ± 4 ^a^	323 ± 19 ^a^	323 ±13 ^a^
Kidneys, mg/mm	47.5 ± 1.8 ^c^	55.2 ± 1.1 ^b^	58.4 ± 3.2 ^ab^	63.6 ± 1.8 ^a^
Spleen, mg/mm	14.6 ± 0.7 ^b^	16.9 ± 0.9 ^b^	15.9 ± 0.6 ^b^	20.2 ± 1.0 ^a^
Plasma alanine transaminase activity, U/L	31.5 ± 6.6	39.5 ± 3.2	47.0 ± 3.0	31.0 ± 2.0
Plasma aspartate transaminase activity, U/L	82.0 ± 11.2	75.5 ± 6.8	72.0 ± 3.0	65.0 ± 4.0

Values are mean ± SEM and *n* = 8 for each group. Mean values within a row with unlike superscript letters are significantly different (a, b, c or d; *p* < 0.05). C, corn starch diet-fed rats; H, high-carbohydrate, high-fat diet-fed rats; HD, high-carbohydrate, high-fat diet-fed rats supplemented with *Oedogonium* grown in ash dam water; HM, high-carbohydrate, high-fat diet-fed rats supplemented with *Oedogonium* grown in treated municipal wastewater.

**Table 3:** Effects of *Oedogonium* grown in ash dam water and treated municipal wastewater on metal composition in the liver.

Metals (mg/kg Dry Weight)	C	H	HD	HM
Al	1.99 ± 0.89	<0.5	0.53 ± 0.24	1.93 ± 0.86
As	4.94 ± 2.21	2.60 ± 1.16	17.34 ± 7.76	2.53 ± 1.13
B	28.20 ± 12.61 ^a^	0.24 ± 0.11 ^b^	11.62 ± 5.20 ^ab^	4.36 ± 1.95 ^ab^
Ba	<0.1	0.08 ± 0.03	0.06 ± 0.03	0.11 ± 0.05
Ca	135 ± 60	129 ± 58	108 ± 48	108 ± 48
Cd	<0.05	<0.05	<0.05	<0.05
Co	0.05 ± 0.02	<0.1	0.21 ± 0.10	0.07 ± 0.03
Cr	0.25 ± 0.11	0.18 ± 0.08	0.16 ± 0.07	0.14 ± 0.06
Cu	15.24 ± 6.82	16.55 ± 7.40	11.92 ± 5.33	13.92 ± 6.23
Fe	781 ± 349	543 ± 243	762 ± 341	526 ± 235
Hg	2.67 ± 1.19	1.75 ± 0.78	1.62 ± 0.72	1.81 ± 0.81
K	10,378 ± 4641	10,416 ± 4658	8622 ± 3856	8197 ± 3666
Mg	717 ± 321	714 ± 319	611 ± 273	655 ± 293
Mn	6.24 ± 2.79	5.49 ± 2.46	5.57 ± 2.49	6.37 ± 2.85
Mo	0.87 ± 0.39	1.03 ± 0.46	1.00 ± 0.45	0.96 ± 0.43
Na	3052 ± 1365	2800 ± 1252	1373 ± 614	1143 ± 511
Ni	0.08 ± 0.03	0.05 ± 0.02	0.08 ± 0.04	0.07 ± 0.03
P	9048 ± 4046	9342 ± 4178	7026 ± 3142	7590 ± 3394
Pb	0.08 ± 0.04	0.07 ± 0.03	0.07 ± 0.03	0.07 ± 0.03
S	5232 ± 2340	5146 ± 2301	4060 ± 1816	4218 ± 1886
Se	1.76 ± 0.79	1.81 ± 0.81	2.99 ± 1.34	2.27 ±1.01
Sr	0.12 ± 0.05	0.18 ± 0.08	0.22 ± 0.10	0.19 ± 0.08
V	0.06 ± 0.03 ^b^	0.09 ± 0.04 ^b^	4.07 ± 1.82 ^a^	0.18 ± 0.08 ^b^
Zn	66.5 ± 29.7	69.7 ± 31.2	57.3 ± 25.6	66.3 ± 29.7

Values are mean ± SEM and *n* = 8 for each group. Mean values within a row with unlike superscript letters are significantly different (a or b; *p* < 0.05). C, corn starch diet-fed rats; H, high-carbohydrate, high-fat diet-fed rats; HD, high-carbohydrate, high-fat diet-fed rats supplemented with *Oedogonium* grown in ash dam water; HM, high-carbohydrate, high-fat diet-fed rats supplemented with *Oedogonium* grown in treated municipal wastewater.

## Data Availability

The original data generated for the study are included in this article.
